# Clinical and Psychosocial Impact of Psychoeducational Groups for Psychosis

**DOI:** 10.1192/j.eurpsy.2024.1492

**Published:** 2024-08-27

**Authors:** F. B. F. Leitão, C. Cunha, J. Loureiro, A. Guedes, M. J. Ribeiro, C. Loureiro, J. Meira, P. Oliveira, J. R. Silva, P. M. Ferreira, A. M. Moreira

**Affiliations:** Serviço de Saúde Mental e Comunitária do Porto Ocidental, Hospital de Magalhães Lemos, Centro Hospitalar Universitário de Santo António, Porto, Portugal

## Abstract

**Introduction:**

Individuals with mental health disorders often lack access to appropriate care, including psychosocial rehabilitation programs, which are considered essential for their recovery. In 2019, as part of the intervention by the *Community and Mental Health Service,* at *Hospital de Magalhães Lemos*, we initiated a psychoeducational group for patients with psychotic spectrum disorders, with the purpose of providing our patients with comprehensive information about their condition and effective management strategies. Our 8-week program consisted of 16 sessions, including icebreaker activities, discussion of certain themes, sharing of experiences and practice of stress management techniques.

**Objectives:**

The aim of this study was to assess and quantify the impact of our 2023 program.

**Methods:**

Out of a total of 20 patients interviewed for our program in 2023, 16 began the program and 12 completed it. The program’s evaluation was based on several assessment tools, including a sociodemographic questionnaire, a knowledge assessment questionnaire, *the Positive and Negative Syndrome Scale (PANSS)*, *the Insight and Treatment Attitudes Questionnaire (ITAC), the World Health Organization Quality of Life (WHOQOL)*, and *the Medication Adherence Rating Scale (MARS).* We also created a health agenda to organize an individual plan of care.

**Results:**

Our findings indicated an improvement in insight and attitudes towards treatment by 8.6%, an enhancement in treatment adherence by 5%, and an increase in knowledge by 11.9%. In terms of quality of life, we observed a slight improvement in the psychological domain by 0.6% and in the social domain by 1.2%. Regarding the impact on psychotic symptomatology, there was an average decrease in 4 points in the negative subscale and in 3 points in the general psychopathology subscale, whereas the positive subscale remained unchanged. None of the patients required hospitalization during this period.

**Conclusions:**

Our study revealed some improvement in nearly all the evaluated parameters. There was an improvement of the therapeutic relationship, which we believe has contributed to lower scores in the negative symptoms and general psychopathology subscale. As for the study limitations, we acknowledge that we will need to expand our sample through additional programs in the next years, to include it in early intervention psychosis programs and to re-evaluate our patients’ outcomes after a more extended follow-up period, particularly if they continue to participate in our monthly mutual support group. Additionally, we must consider potential study biases, including the subjectivity of *PANSS* evaluations and the influence of other confounding factors, such as changes in treatment regimens during the program.

**Disclosure of Interest:**

None Declared

